# Supplementary material for: Ultraviolet astronomical spectrograph calibration with laser frequency combs from nanophotonic lithium niobate waveguides
Source: Nat Commun. 2024 Sep 2;15:7614. doi: 10.1038/s41467-024-51560-x (PMC11369296; doi:10.1038/s41467-024-51560-x)
Supplement: Supplementary file 1 — Supplementary Information [file 41467_2024_51560_MOESM1_ESM.pdf]

Ultraviolet astronomical spectrograph calibration with laser frequency combs from nanophotonic lithium niobate waveguides

Supplementary Information

Markus Ludwig,[1,*] Furkan Ayhan,[2,*] Tobias M. Schmidt,[3,*]
Thibault Wildi,[1] Thibault Voumard,[1] Roman Blum,[4] Zhichao Ye,[5] Fuchuan Lei,[5]
François Wildi,[3] Francesco Pepe,[3] Mahmoud A. Gaafar,[1] Ewelina Obrzud,[4] Davide Grassani,[4]
Olivia Hefti,[4] Sylvain Karlen,[4] Steve Lecomte,[4] François Moreau,[6] Bruno Chazelas,[3] Rico Sottile,[6]
Victor Torres-Company,[5] Victor Brasch,[7] Luis G. Villanueva,[2] François Bouchy,[3] Tobias Herr[1,8,**]

[1]Deutsches Elektronen-Synchrotron DESY, Notkestr. 85, 22607 Hamburg, Germany
[2]École Polytechnique Fédérale de Lausanne (EPFL), 1015 Lausanne, Switzerland
[3] Observatoire de Genève, Département d'Astronomie, Université de Genève, Chemin Pegasi 51b, 1290 Versoix, Switzerland
[4] Swiss Center for Electronics and Microtechnology (CSEM), 2000 Neuchâtel, Switzerland
[5] Department of Microtechnology and Nanoscience, Chalmers University of Technology, 41296 Gothenburg, Sweden
[6] Observatoire de Haute-Provence, CNRS, Université d'Aix-Marseille, 04870 Saint-Michel-l'Observatoire, France
[7] Q.ANT GmbH, Handwerkstraße 29, 70565 Stuttgart, Germany
[8]Physics Department, Universität Hamburg UHH, Luruper Chaussee 149, 22607 Hamburg, Germany
[*]These authors contributed equally.
[**]tobias.herr@desy.de

1 Linewidth and background in the harmonic frequency comb

The lineshape of a comb line is defined by its phase noise spectrum. Phase noise may broaden the linewidth or may contribute to a broadband background signal. Both linewidth and background have important implications for astrocombs and are discussed below.

1.1 Phase noise and comb lineshape

Both ν_0 and f_{rep}, which are the defining frequencies of the comb (Eq. 1, main text), exhibit non-zero phase noise described by their (single-sided) phase noise power spectral densities (PSDs) $S_\phi^{\nu_0}(f)$ and $S_\phi^{f_{\text{rep}}}(f)$, where f is the offset frequency from the respective carrier wave. The resulting phase noise of the comb lines is

$$S_\phi^{\nu_{m,n}} = m^2 S_\phi^{\nu_0} + n^2 S_\phi^{f_{\text{rep}}} , \tag{1}$$

where $m = 1, 2, ..$ indicates the harmonic and $n = \pm 1, \pm 2, ..$ the line index relative to the center of the harmonic. The second term usually limits the useful span (i.e., a maximal $|n|$) of an electro-optic comb generator. To increase the useful span, noise-filtering can be applied to suppress the emergence of a strong background signal as well as linewidth broadening [1].

Creating broadband comb spectra via cascaded $\chi^{(2)}$-harmonics, rather than $\chi^{(3)}$-based broadening around ν_0, shows distinct noise properties as frequencies ν much higher than ν_0 can be reached through lines in a harmonic m while keeping $n << (\nu_{m,n} - \nu_0)/f_{\text{rep}}$. Neglecting the small impact of $m^2 S_{\phi\phi}^{\nu_0}$ (as $m << n$), the phase noise of comb lines of frequency $\nu_{m,n}$ that are part of the mth harmonic comb does not grow in function of their frequency difference from ν_0, but only with their distance from the mth harmonics center frequency $\nu_{m,0}$. This permits generating UV astrocombs in a limited span around the fourth harmonic center frequency $\nu_{4,0}$ from a near-infrared electro-optic comb generator without noise-filtering; wider spectra around the harmonics will still require noise filtering. The different noise properties of $\chi^{(2)}$ and $\chi^{(3)}$-based spectra are illustrated in Supplementary Fig. 1.

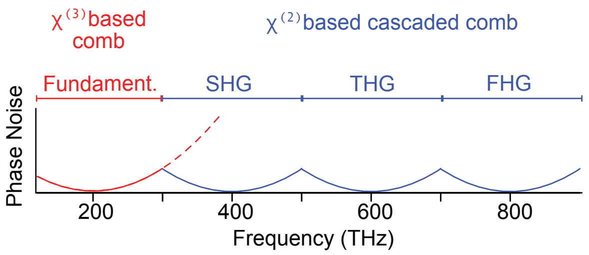

Supplementary Figure 1 | **Phase noise** in a traditional $\chi^{(3)}$-based comb compared to the phase noise a $\chi^{(2)}$-based comb generated through cascaded harmonics..

Based on the phase noise $S_\phi^{f_\mathrm{rep}}$ we can (numerically) estimate the lineshape $S_n(\nu_\mathrm{rel})$ of a comb line that is n lines away from the center of the harmonic by computing the Fourier transform (ν_rel is the optical frequency relative to the center of the optical line)

$$S_n(\nu_\mathrm{rel}) = 2\int_{-\infty}^{\infty} \Gamma(\tau)\exp\left[-i2\pi\nu_\mathrm{rel}\tau\right]\,\mathrm{d}\tau \tag{2}$$

of the normalized autocorrelation function [2]

$$\Gamma(\tau) = \exp\left[-2\int_0^{\infty} n^2 S_\phi^{f_\mathrm{rep}}(f)\sin^2(\pi f\tau)\,\mathrm{d}f\right]\,. \tag{3}$$

1.2 Experimental observation of line contrast and comparison with numeric model

An astronomical spectrograph cannot resolve the lineshapes of the comb lines but instead observes the convolution of the comb spectrum with the spectrograph's instrumental line-spread function (LSF). Depending on the lineshape of the comb lines (linewidth and background), the spacing of the comb lines and the LSF, comb lines may be observed by the spectrograph with a reduced level of contrast. Based on the discussion in Supplementary Section 1.1 one would expect that the observed line contrast reduces for larger $|n|$ due to the increase of phase noise (causing increased background and linewidth). This is indeed observed in the experimental data. Supplementary Figure 2a,b, shows the line contrast (extracted from main text Figure 3 as the peak-valley difference divided by the peak height) in dependence of the line index n relative to the spectral centers of the third and fourth harmonics. As expected, the contrast is highest in the center of the harmonics and decreases with spectral distance from the harmonic center. Under the assumption that in the center of the harmonics the lines are narrow and there is negligible background, we can estimate the full-width-at-half maximum (FWHM) of the instrumental line-spread function which we assume to be of Gaussian shape. We find ≈ 7.9 GHz for the FWHM in the spectral region of the third harmonic and 9.8 GHz to 10.8 GHz in the spectral region of the fourth harmonic. Assuming that the PSD of the repetition rate phase noise can be approximated at noise frequencies up to 30 MHz by the phase noise of our microwave synthesizer and above 30 MHz exhibits a white phase noise floor of -165 dBc/Hz (see 2c), we derive the lineshapes according to Supplementary Section 1.1 and perform the convolution with the estimated instrumental line-spread functions. These model results are overlayed in the Supplementary Figure 2a,b and show good qualitative agreement with the observed data. For clarity we note that the experimental noise floor in $S_\phi^{f_\mathrm{rep}}$ may not only stem from the microwave source but also emerge from amplified spontaneous emission (ASE) in the erbium-doped fiber amplifiers or other noise sources. To better illustrate the underlying comb lineshapes (prior to convolution), Supplementary Figure 2d shows their characteristic shapes for different values of n. Panels e and f show the linewidth at different levels as well as the relative power contained in the narrow line and in the flat background as function of n. The background power is determined by multiplying the background level in the optical PSD with the comb line spacing f_rep. Subtracting the background power from the total power yields the line power.

1.3 Comparison of linewidth and line/background power in $\chi^{(2)}$- and $\chi^{(3)}$-based combs

To provide additional information, also with regard to future implementations of UV electro-optic astrocombs, we have added a detailed comparison of $\chi^{(2)}$- and $\chi^{(3)}$-based comb generation in Supplementary Figure 3. This figure shows in the left column (panels a,b) the conventional $\chi^{(3)}$-based approach and in the right column (panels c,d) the approach based on $\chi^{(2)}$ processes. In both cases we assume the repetition rate phase noise as shown in Figure 2c, where for the $\chi^{(3)}$-based approach we also show a scenario with a noise floor at -185 dBc/Hz (such low noise floor could be achieved when the synthesizer outputs at least 11 dBm of microwave power (thermal noise floor at -174 dBm/Hz); higher power

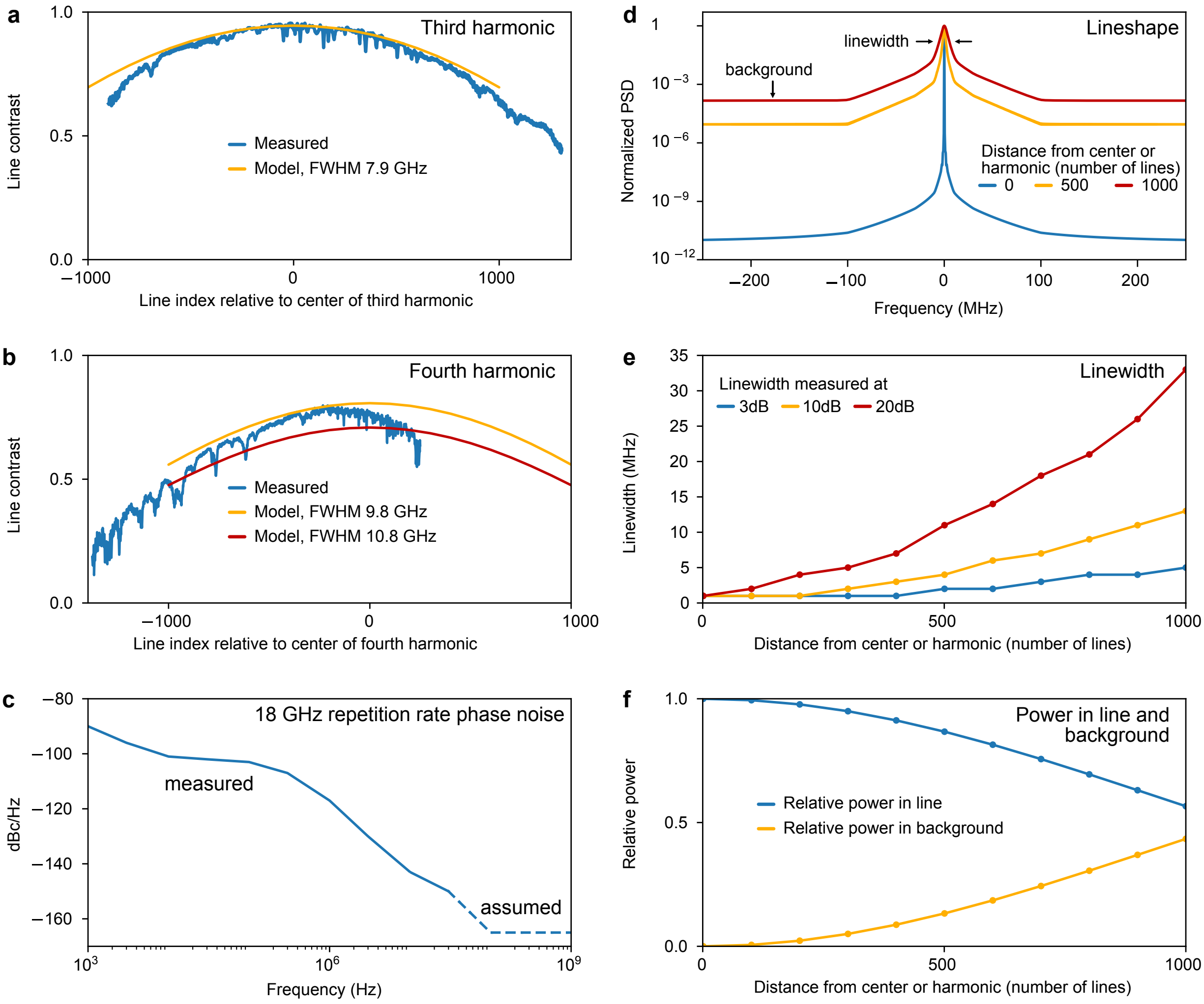

Supplementary Figure 2 | Contrast and lineshape. a, Measured line contrast of the third harmonic and predicted contrast based on the model described in the text for an instrumental line-spread function with a full-width-at-half-maximum (FWHM) of 7.9 GHz. **b,** Same as in panel a, however, for the fourth harmonic and assuming an instrumental line-spread function with FWHM of 9.8 GHz and 10.8 GHz. **c,** Power spectral density $S_\phi^{f_{\rm rep}}$ of the repetition rate phase noise for our model, based on the measured phase noise of the microwave synthesizer driving the electro-optic comb up to 30 MHz frequency and assumed phase noise above 30 MHz (see text for details). **d,** Comb lineshape (normalized PSD) for the phase noise in panel c for different distances from the center of a harmonic. **e,** 3dB, 10dB and 20dB linewidth of the comb lines in dependence of their distance from the center of a harmonic. **f,** Relative power contained in the comb line and in the flat background (see text for details) in dependence of their distance from the center of a harmonic.

is needed when additional noise, e.g. from microwave amplification, is relevant). As a comparison between panels a and c shows, the width of the comb lines generated in the $\chi^{(2)}$-based approach can be well below those generated in a $\chi^{(3)}$-based approach. Over the wavelength range utilized in our work, and with the parameters assumed here, the $\chi^{(2)}$-generated lines, even without noise suppression, are expected to be narrower than those one would expect from a $\chi^{(3)}$-based approach with a noise suppression filter (Here we assume a noise filter of 50 MHz width). Figure 3 panels b and c compare the relative power that is contained in the line and in the flat background. The spectral interval utilized in our work is highlighted in panels c and d, indicating a sufficiently low contribution of the background so that the comb lines can be observed. Noise filtering can provide effective means of suppressing the background flux both for the $\chi^{(3)}$-based approach as well as for extending the $\chi^{(2)}$-based approach to wider bandwidth.

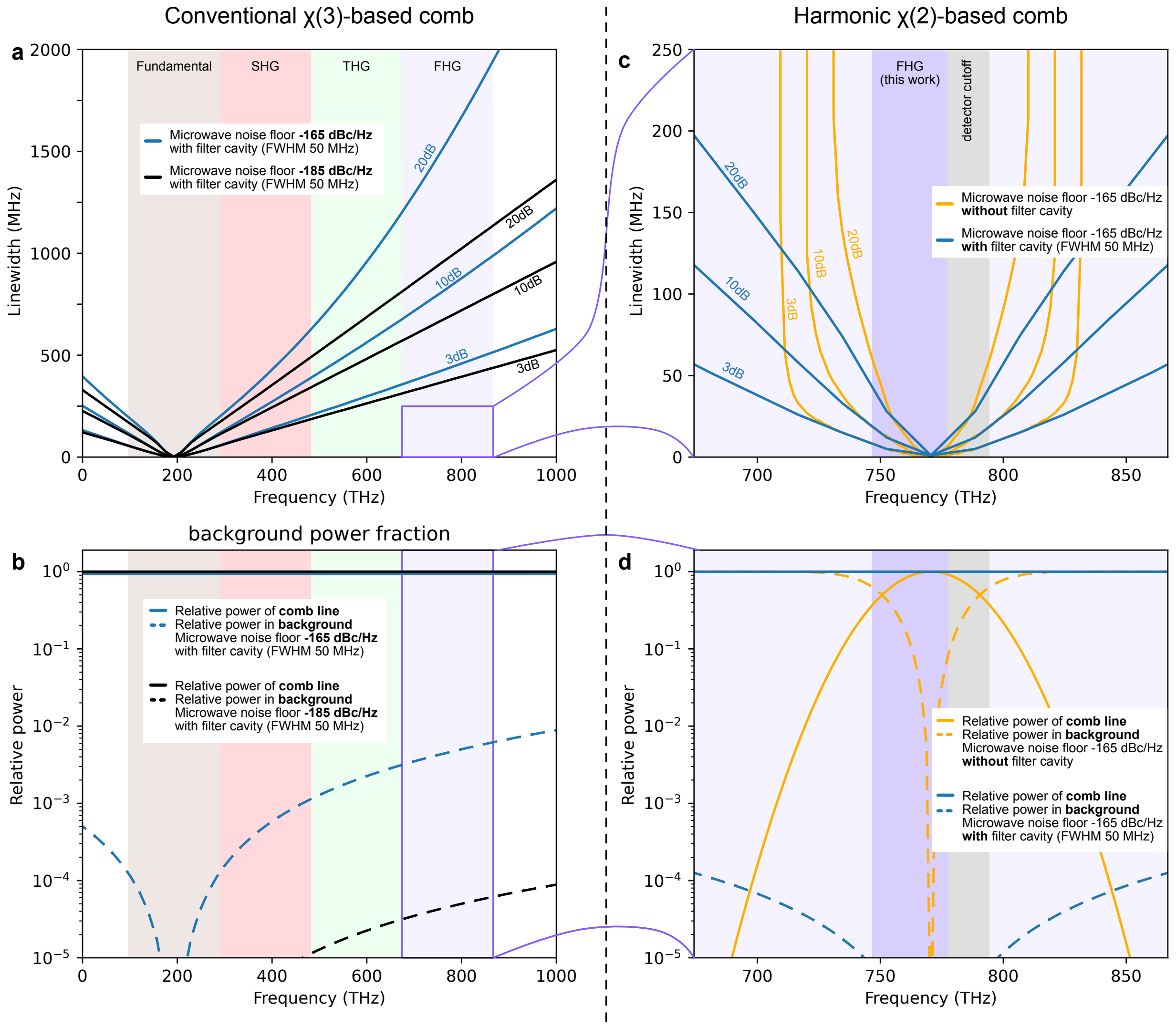

Supplementary Figure 3 | Comparison of key characteristics in $\chi^{(2)}$- and $\chi^{(3)}$-based electro-optic combs, a, 3dB, 10dB and 20dB linewidth for a $\chi^{(3)}$-based electro-optic comb with cavity noise supression (Full-width-at-half-maximum, FWHM, 50 MHz). The microwave phase noise is assumed to be as in Supplementary Figure 2 with a noise floor of -165 dBc/Hz and -185 dBc/Hz, respectively. The color-coded background indicates the spectral domains of fundamental comb, second, third and fourth harmonic generation (SHG, THG, FHG) **b,** Relative power in the flat background and in the comb line for the conditions as in panel a. **c,** 3dB, 10dB and 20dB linewidth for a $\chi^{(2)}$-based electro-optic comb around the center of the fourth harmonic with and without a noise filter cavity and a microwave phase noise floor of -165 dBc/Hz. The spectral range used in this work and the detector cutoff are indicated. **d,** Relative power in the flat background and in the comb line for the conditions as in panel c.

2 Fabrication of periodically-poled lithium niobate waveguides

Lithium niobate waveguides for UV comb generation are fabricated on 16-by-16 mm^2 chips diced from a 100 mm wafer (NANOLN) with 800 nm-thick x-cut $LiNbO_3$ layer on 3 µm SiO_2 and bulk Si substrate (Supplementary Figure 4a). To ensure precise alignment of the waveguides and poling electrodes in subsequent lithography steps to the crystal axes of the lithium niobate, platinum (Pt) alignment marks are patterned on wafer scale before dicing. This is done with electron-beam lithography (EBL), electron-beam evaporation (EBE) and a lift-off process.

First, chromium (Cr) poling electrodes are patterned via EBL, EBE and lift-off using MMA/PMMA bilayer resist (Supplementary Figure 4b). Electrodes are patterned such that the applied electric field across the electrodes is along the z-axis of lithium niobate (Supplementary Figure 4c). Ferroelectric domain inversion is performed by applying a high-voltage (HV) field accross the electrodes in two stages: First, a *pre-poling* signal is repeated three times to help nucleation of domains closer to positive (V+) electrode (Supplementary Figure 5a). Secondly, a higher field (30 V/µm), followed by a slow decay is applied to propagate the iniated domains to the ground (GND) electrode (Supplementary Figure 5b). After poling, samples are inspected under a scannning electron microscope (SEM) (Supplementary Figure

5c) before removing the electrodes with wet etching (Supplementary Figure 4d).

Before waveguide fabrication, a Cr layer is deposited using EBE to be used as a hard mask for etching lithium niobate. Later, waveguides are patterned with EBL (Supplementary Figure 4e). The Cr hard mask is etched using argon (Ar) ion-beam etching (IBE) (Supplementary Figure 4f). Subsequently, the lithium niobate layer is fully etched with reactive-ion etching (RIE) with fluorine chemistry (CHF_3/Ar) (Supplementary Figure 4g). After etching, the remaining Cr hard mask is removed and the structures are cleaned with wet etching.

Lastly, samples are cladded with a 3 µm-thick SiO_2 layer, deposited via chemical vapor deposition (CVD) (Supplementary Figure 4h). Finally, waveguide facets are defined by deep etching of SiO_2, $LiNbO_3$ and Si layers to ensure better coupling to the waveguides.

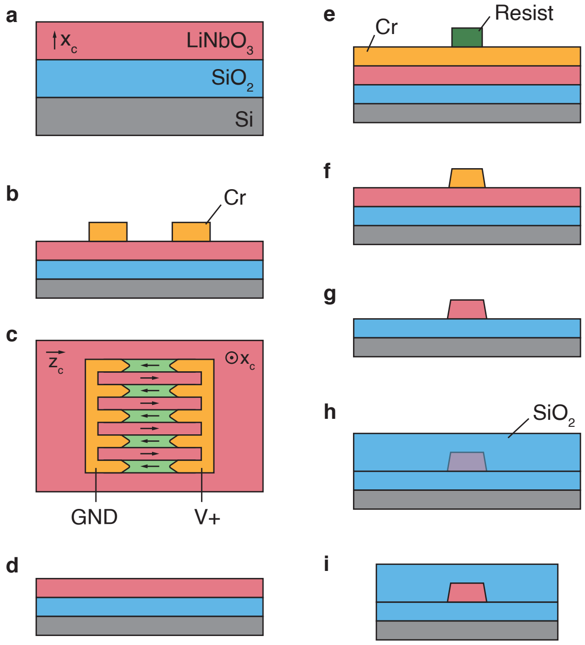

Supplementary Figure 4 | Fabrication of periodically-poled lithium niobate wavegeuides. a, LNOI substrate with 800 nm x-cut $LiNbO_3$, 3 µm SiO_2 and bulk Si. **b,** Deposition of Cr electrodes for periodic poling with electron-beam lithography, electron-beam evaporation and lift-off. **c,** Periodic poling with a high-voltage source. Sample is illustrated from top. **d,** Removal of the Cr electrodes after periodic poling. **e,** Cr hard mask deposition with electron-beam evaporation and patterning of the waveguides with electron-beam lithography. **f,** Ar ion-beam etching of the Cr hard mask. **g,** Fluorine reactive-ion etching of the $LiNbO_3$. **h,** SiO_2 cladding deposition with chemical vapor deposition. **i,** Series of reactive ion etching steps to etch SiO_2, $LiNbO_3$ and Si layers to reveal waveguide facets.

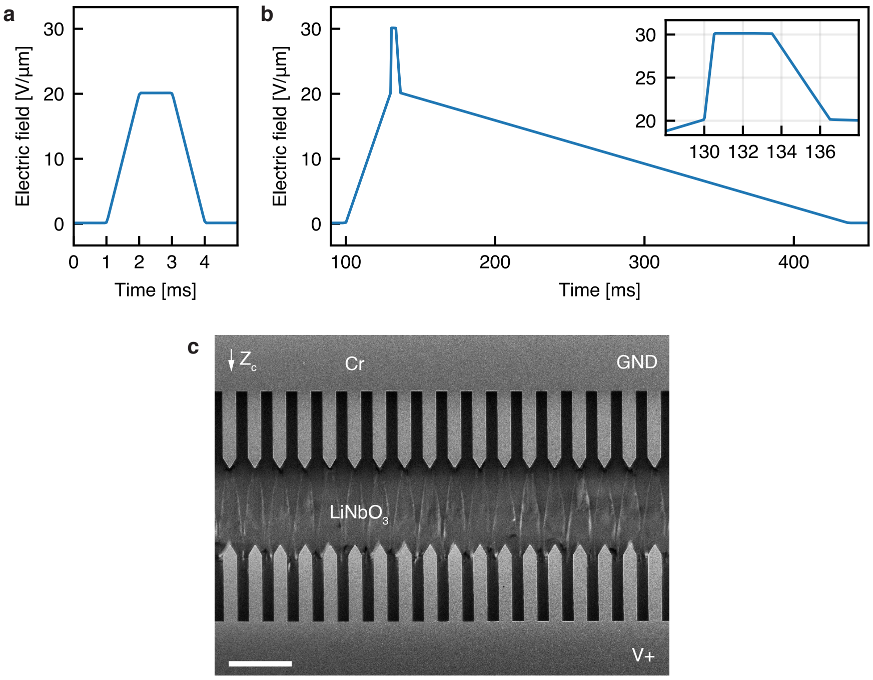

Supplementary Figure 5 | **Periodic poling of the lithium niobate waveguides**. **a,** One of three high-voltage *pre-poling* signals applied to electrodes for periodic poling. **b,** High-voltage *main* signal applied for poling. Inset shows the detailed section of the peak poling signal. **c,** SEM image of the poled lithium niobate sample. Scale bar is 10 µm.

3 Broadband spectrum

To explore the potential of extending the spectral coverage in future work, we configure the 18 GHz electro-optic comb to provide a broadband pulse with an auto-correlation trace as in Figure 6a. This is achieved via a combination of highly-nonlinear bandwidth generating fiber and compression fiber, similar to [3]. With this input pulse we generate the broadband spectrum of overlapping harmonics as shown in Figure 6b.

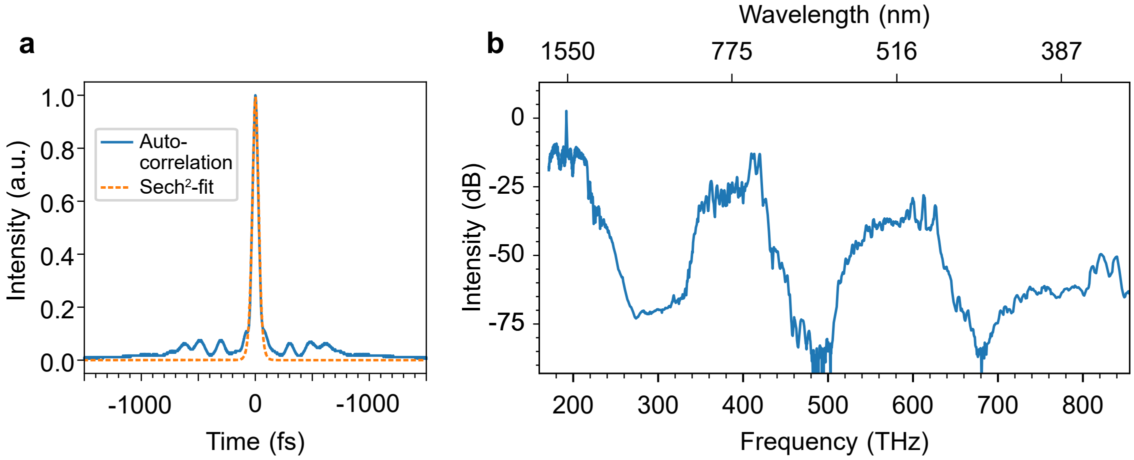

Supplementary Figure 6 | **Broadband spectrum of an 18 GHz comb.** **a,** Auto-correlation trace and pulse duration based on a sech^2-fit, retrieving a pulse duration of 38 fs (full width at half maximum of the intensity transient). **b,** Resulting spectrum with overlapping harmonics recorded on a grating based optical spectrum analyzer (OSA, model Yokogawa AQ6374).

4 Power consumption and nonlinear conversion efficiency

4.1 Power consumption

The energy consumption of the electro-optic comb generator is dominated by the multi-W erbium-doped fiber amplifier (EDFA) and the microwave electronics. Assuming a 10% wall-plug efficiency the EDFA will approximately consume 35 W of electrical power. The microwave synthesizer consumes under maximal load, depending on the specific hardware, ca. 80-130 W of electrical power, the microwave amplifiers jointly ca. 50 W of electrical power. In sum this amounts to approximately 200 W of electrical power consumption, about the same as a standard desktop computer. In case of the microresonator-based approach, one can subtract the power consumption of the microwave source and microwave amplifier, resulting in a power consumption of approximately 35 W. The power consumption of our mode-locked laser is below 15 W. However, considering the ∼200-times lower repetition rate, the energy consumption per pulse is not better than of the electro-optic comb, although it reaches higher per pulse energy.

4.2 Nonlinear conversion efficiency

To derive the conversion efficiency of the fundamental comb into the harmonics, the experimental spectra shown in Figure 2g in the main text complemented by additional spectra at different pulse energies were integrated over the respective spectral ranges of the harmonic orders. The left panel of Supplementary Figure 7 shows the resulting output power levels of the fundamental and the harmonics up to the fourth order as a function of on-chip input pulse energy. The log-log scale clearly reveals that the slopes increase with harmonic order. Consistently, also the pulse energy threshold to cross the noise floor of the spectrum analyzer increases with the harmonic order. The fact that the power in the fundamental saturates beyond 40 pJ and eventually even drops can be attributed both to an increasing depletion into the harmonics and additional spectral broadening of the fundamental which eventually exceeds the range detected by the spectrum analyzer (1750 nm). The right panel of Supplementary Figure 7 shows the conversion efficiency, i.e., the ratio of the power contained in the respective harmonic orders vs. the power in the fundamental comb. A conversion efficiency well exceeding 0.1 % is achieved for the fourth harmonic.

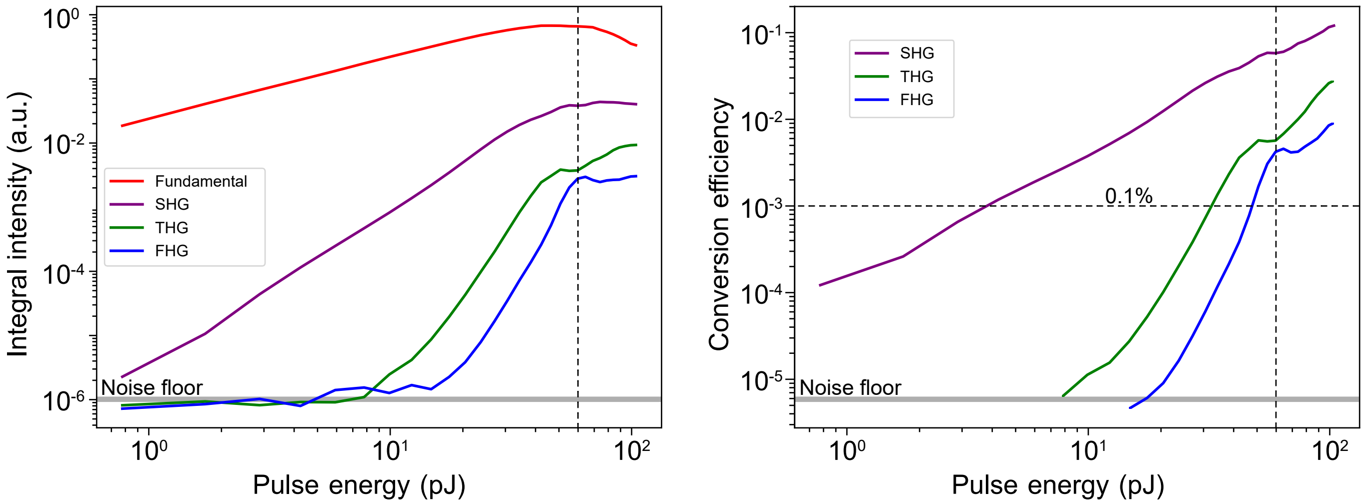

Supplementary Figure 7 | Conversion efficiencies of the fundamental spectrum into the harmonics. Left: Integrated intensity contained in the Fundamental spectral domain and the domains of second, third and fourth harmonic generation (SHG THG FHG). The vertical dashed line indicates a maximum on-chip input pulse energy of roughly 60 pJ beyond which the power in the fourth harmonic saturates. **Right:** Corresponding conversion efficiency.

5 UV irradiation test of a lithium niobate waveguide

To assess the susceptibility of our waveguides to UV-induced damage, we performed an additional test, where we coupled a 405 nm laser diode to a lithium niobate waveguide (1-1.5 mW of coupled power). To perform this test, we fabricated dedicated lithium niobate waveguides that are not straight but have an S-bend on the waveguide such that input and output are offset from each other (i.e. offset in the direction orthogonal to the direction of the incoming light). This configuration enables accurate measurements of the coupled intensities without picking up stray light that has not propagated through the waveguide. Moreover, a narrow waveguide with a width of 600 nm was chosen to enhance the intensity. Over 12 hours we delivered more than 60 J optical energy through the waveguide, which would correspond to more than one week of UV exposure in astrocomb operation (assuming a UV power level of 100 μW). We did not observe a noticeable degradation of the UV or infrared transmission through the waveguide within the precision of the measurement. Although the test waveguides were unpoled and would hence not have been suitable for spectrograph calibration, we also performed a supercontinuum generation experiment before and after UV irradiation resulting essentially in the same spectra, as shown in Supplementary Figure 8

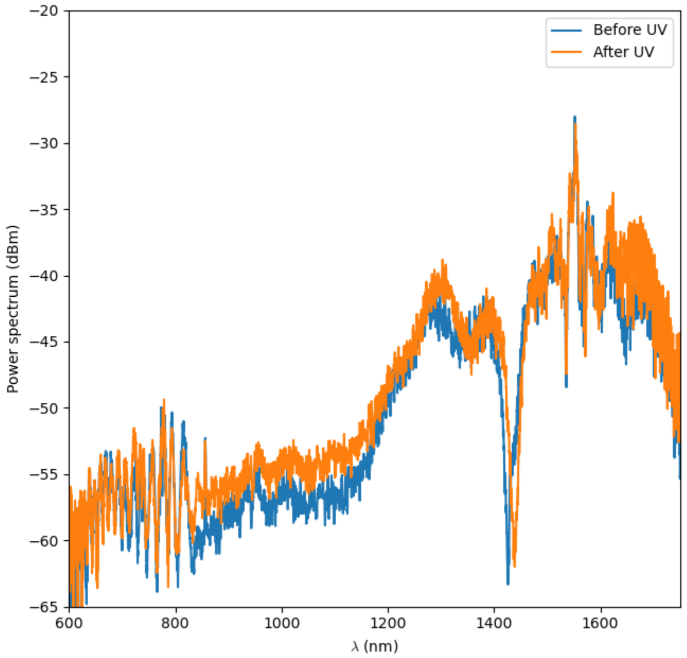

Supplementary Figure 8 | Supercontinuum before and after UV irradiation. Spectrum of a supercontinuum generated in the lithium niobate waveguide before (blue) and after (orange) UV irradiation. The mode-locked laser driving the supercontinuum has an average power of 73 mW, a pulse duration of 125 fs and repetition rate of 40 MHz.

References

1. Beha, K. *et al.* Electronic Synthesis of Light. *Optica* **4,** 406–411 (2017).
2. Domenico, G. D., Schilt, S. & Thomann, P. Simple Approach to the Relation between Laser Frequency Noise and Laser Line Shape. *Applied Optics* **49,** 4801–4807 (2010).
3. Obrzud, E. *et al.* Broadband Near-Infrared Astronomical Spectrometer Calibration and on-Sky Validation with an Electro-Optic Laser Frequency Comb. *Optics Express* **26,** 34830–34841 (2018).
